# Beneficial Effects of Opioid Rotation to Buprenorphine/Naloxone on Opioid Misuse, Craving, Mental Health, and Pain Control in Chronic Non-Cancer Pain Patients with Opioid Use Disorder

**DOI:** 10.3390/jcm10163727

**Published:** 2021-08-21

**Authors:** Arnt F. A. Schellekens, Stijn E. Veldman, Eka S. D. Suranto, Steffie M. van Rijswijk, Selina E. I. van der Wal, Aart H. Schene, Marleen H. C. T. van Beek

**Affiliations:** 1 Department of Psychiatry, Radboud University Medical Center, Geert Grooteplein Zuid 10, 6525 GA Nijmegen, The Netherlands; Arnt.Schellekens@radboudumc.nl (A.F.A.S.); ekayanisuranto@hotmail.com (E.S.D.S.); steffievanrijswijk1987@hotmail.com (S.M.v.R.); aart.schene@radboudumc.nl (A.H.S.); Marleen.vanBeek@radboudumc.nl (M.H.C.T.v.B.); 2 Nijmegen Institute for Scientist-Practitioners in Addiction (NISPA), 6500 HE Nijmegen, The Netherlands; 3 Donders Institute for Brain Cognition and Behavior, Radboud University Nijmegen, 6525 AJ Nijmegen, The Netherlands; 4 Department of Anesthesiology Pain and Palliative Medicine, Radboud University Medical Center, 6525 GA Nijmegen, The Netherlands; Selina.vanderWal@radboudumc.nl

**Keywords:** opioid use disorder, chronic non-cancer pain, buprenorphine/naloxone, opioid misuse, craving, depression, anxiety, stress, pain

## Abstract

Patients with chronic non-cancer pain (CNCP) often use opioids for long periods of time. This may lead to opioid use disorder (OUD) and psychiatric symptoms: mainly depression and anxiety. The current study investigated the effect of buprenorphine/naloxone (BuNa) rotation on opioid misuse, craving, psychiatric symptoms and pain in patients with CNCP and OUD. Forty-three participants with CNCP and OUD were converted from a full mu-receptor agonist opioid (mean morphine equivalent dose: 328.3 mg) to BuNa, in an inpatient setting. Opioid misuse, craving, co-occurring psychiatric symptoms, and pain perception were determined at baseline and after a two-month follow-up, using the following self-report questionnaires: Current Opioid Misuse Measurement (COMM), Visual Analog Scale (VAS-craving and VAS-pain) and Depression, Anxiety and Stress Scale (DASS), respectively. VAS-craving and VAS-pain were also determined immediately after conversion. A total of 37 participants completed the protocol. The mean COMM decreased from 17.1 to 6.7 (F = 36.5; *p* < 0.000), the mean VAS-craving decreased from 39.3 to 5.3 (−86.6%; F = 26.5, *p* < 0.000), the mean DASS decreased from 12.1 to 6.6 (F = 56.3, *p* < 0.000), and the mean VAS-pain decreased from 51.3 to 37.2 (−27.4%, F = 3.3; *p* = 0.043). Rotation to BuNa in patients with CNCP and OUD was accompanied by reductions in (i) opioid misuse, (ii) opioid craving, (iii) the severity of co-occurring psychiatric symptoms, and (iv) self-reported pain. BuNa as opioid agonist treatment may therefore be a beneficial strategy in CNCP patients with OUD. The limited sample size and the observational nature of this study underline the need for the replication of the current findings in large-scale, controlled studies.

## 1. Introduction

Worldwide there has been a marked increase in opioid prescriptions for chronic non-cancer pain (CNCP) since the mid-1990s [1,2]. Particularly, the United States (US) faced a large increase in opioid prescriptions, leading to prescription opioid use in 17.4% of the general population in the US, in 2017 [3].

Despite the effectiveness of opioids as analgesics in severe acute pain, there is limited evidence for the long-term analgesic effects of opioids [4,5]. In addition, long-term opioid use is associated with numerous adverse effects, including constipation, respiratory depressions, sedation, reduced concentration, opioid use disorder (OUD) and opioid-related mortality [6,7,8,9]. In addition, both chronic pain and long-term opioid use are associated with the development of psychiatric comorbidities, including depression and anxiety, and reduced quality of life [10,11,12]. Physical dependence may develop rapidly after the initiation of opioid use, leading to a tolerance for the analgesic effects of opioids, and withdrawal when opioids are not taken. Furthermore, both opioid-induced hyperalgesia, characterized by an increased sensitivity to pain induced by full mu-receptor agonists, and tolerance for the analgesic effects of full mu-opioid receptor agonists, may contribute to a desire for increasing opioid doses [13,14]. Patients may subsequently develop OUD, including the continued use despite many negative health consequences, the loss of control of opioid use, and cravings for opioids [15]. A systematic review found a point-prevalence of addiction of 8–12% among patients with CNCP [16]. In addition, meta-analysis found a pooled incidence of OUD in approximately 4.7% of patients using opioids for pain relief [17].

The combination of prescription OUD and CNCP poses a clinical challenge since, on the one hand OUD requires the tapering of opioids, while on the other hand this might temporarily increase pain and craving, hindering the successful detoxification of opioids [18,19,20,21]. An alternative strategy for these patients might be opioid agonist treatment (OAT) with a long-acting mu-opioid receptor agonist, since long-acting opioids might not only stabilize opioid use, but simultaneously provide pain relief. Indeed, some studies investigated the potential of methadone as an OAT in patients with prescription OUD and CNCP, showing long-lasting improvement in pain control [22,23,24].

Buprenorphine OAT, optionally provided as a combination therapy with naloxone, might be of specific interest for the treatment of patients with prescription OUD and co-occurring CNCP, given its pharmacological profile [19,25]. Buprenorphine is a high-affinity partial mu-opioid receptor agonist. Several studies suggest that buprenorphine has similar equi-analgesic properties as full mu-opioid receptor agonists, like morphine and transdermal fentanyl [26]. Studies on the effectiveness of buprenorphine rotation in patients with CNCP and OUD suggested a positive effect on pain [23,27,28,29,30,31]. However, these studies had a small sample size [23,31], were retrospective in nature [27,28,30], or participants could self-administer additional oxycodone [29]. Additionally, buprenorphine has a lower risk of respiratory depression, sedation and overdose [32,33]. Furthermore, the dissociation rate of buprenorphine is slow, resulting in a long duration of action [32]. OAT with buprenorphine, commonly taken in combination with naltrexone (BuNa), has repeatedly been associated with a decrease in craving over time in patients with OUD without comorbid CNCP [34,35,36,37,38,39]. To our knowledge, only one study investigated the effects of BuNa on craving in patients with iatrogenic OUD and co-morbid CNCP [23]. However, this study had a small sample size (*n* = 19) and did not distinguish between the conversion to methadone or BuNa. Finally, buprenorphine is a full kappa-opioid receptor antagonist, which has been associated with antidepressant and anxiolytic effects [40,41,42,43]. In line with this, there is an indication that buprenorphine has anti-depressant abilities [44,45,46]. These effects have also been found in patients with OUD [47,48,49,50,51] and patients with CNCP [23].

Since the effects of BuNa OAT on opioid misuse, craving and psychiatric outcomes have hardly been studied in CNCP patients with co-occurring OUD, the aim of the current study was to explore the effectiveness of BuNa in patients with CNCP and OUD. The primary objective was to study the effectiveness of BuNa in reducing opioid misuse and craving. Secondary objectives included exploring the effects of BuNa on the severity of co-occurring psychiatric symptoms and self-reported pain. Specifically, we tested the hypotheses that opioid rotation to BuNa: (i) reduced current opioid misuse, (ii) reduced opioid craving, (iii) improved psychiatric symptoms, and (iv) reduced self-reported pain.

## 2. Materials and Methods

### 2.1. Design

In this prospective, open-label, observational study, patients with CNCP and OUD were rotated in an inpatient setting from their full mu-opioid receptor agonist to BuNa. The regional medical ethical board approved this study (2015–1551) and all participants gave written informed consent.

### 2.2. Participants

Participants (*n* = 43) with CNCP, who fulfilled criteria of chronic pain syndrome (longer than 6 months) and had a co-occurring prescription OUD according to Diagnostic and Statistical Manual of Mental Disorders 5 (DSM-5) [52,53], were referred to the Department of Psychiatry of the Radboud University Medical Center (UMC) by their pain specialist or general practitioner. The participants were volunteers seeking treatment for their OUD and their admission was solely aimed at the BuNa rotation. Besides CNCP and OUD, other inclusion criteria were: aged between 18–65 years, used opioids for at least one year and an oral morphine equivalent (OME) dose exceeding 90 mg per day. Participants with contraindications for BuNa (i.e., severe respiratory insufficiency, hepatic insufficiency) and severe acute psychiatric comorbidity (e.g., acute psychosis, acute mania or severe depression with suicidal ideation), were excluded. The participants were screened for severe acute psychiatry at the outpatient Department of Dsychiatry at Radboud UMC, based on clincal judgement by a trained physician assistant and experienced addiction psychiatrist. None of the referred participants met the exclusion criteria. Data were collected between 2017–2019.

### 2.3. Intervention

The rotation from full agonist opioids to BuNa took place in an inpatient Medical Psychiatric Unit. In the first week of admission, long-acting morphine mimetics (e.g., fentanyl and oxycodone), if used, were switched to dose-equivalent, short-acting oxycodone. On the first morning of the consecutive week all short-acting oxycodone was stopped. After complaints of withdrawal arose, guided by the objective withdrawal scale (OOS) and subjective withdrawal scale (SOS) [54], the first dose of BuNa was given. Participants started with 4/1 mg BuNa. Hereafter, the BuNa dose was subsequently titrated with 2/0.5 mg per 4 hours, based on subjective and objective withdrawal scores and pain perception, to a maximum of 24/6 mg BuNa on day 1. The next day, participants received the entire dose of the first day, divided in maximum three dosages, with additional BuNa if needed. Hereafter, the BuNa dose was, similar to the previous day, titrated to a maximum of 36/9 mg per day. The final dose scheme was determined in seven days and the participants stayed on this scheme until the follow-up after two months, unless dose adaptations were necessary, e.g., due to severe side effects. Additional medication was prescribed as needed to counteract withdrawal symptoms (clonidine, metoclopramide, and loperamide) and pain (paracetamol, ibuprofen), in line with Dutch detoxification guidelines and the guidelines of the American Society of Addiction Medicine [54,55]. In addition, during the inpatient setting, the participants could participate in daily activities, consisting of routine clinical care, offered to all patients admitted to our psychiatric unit, including a morning opening meeting, a daily walk through the park and creative activities. After discharge, no additional treatments or changes regarding medication were allowed.

### 2.4. Instruments

Socio-demographic data (sex, age, ethnicity, marital status, years of education, and employment status) were collected on admission, as well as some baseline clinical data (kind of opioid used, years of opioid used, OME, use non-opioid analgesics, other comedication, use of other psychoactive substances, pain type, and psychiatric history). Screening for current psychiatric disorders was performed using the Mini International Neuropsychiatric Interview (MINI-Plus) [56,57] by trained clinicians. The MINI-plus was a structured and standardized diagnostic interview used to determine the most common psychiatric disorders according to axis I DSM-IV-TR and the International Classification of Diseases and Related Health Problems (ICD-10) [56,57,58]. It is widely used both clinically and in research, and has been well-validated [56,57].

### 2.5. Opioid Misuse

Opioid misuse was assessed using the Current Opioid Misuse Measure (COMM) [59,60,61]. This was a self-report measure of aberrant opioid use in the past month. The COMM consisted of 17 items and was created to monitor potential opioid misuse during opioid treatment. All items were scored between 0 and 4, and subsequently summed into a total score. Total scores above 9 were considered positive for opioid misuse. It was shown to be a reliable and valid instrument to detect opioid misuse in CNCP patients [60].

Furthermore, opioid craving was assessed as an index of opioid misuse severity, using a visual analog scale (VAS-craving). The VAS-craving was a quantitative measurement where the participant quantifies their current state of craving by marking a point on a scale form 0–100, with 0 as no craving at all and 100 as the most severe craving imaginable [62]. VAS-craving measurement was commonly used in addiction medicine to monitor craving severity [62].

### 2.6. Psychiatric Symptoms

Psychiatric symptom severity was assessed using the Depression, Anxiety and Stress Scale (DASS) [63]. The DASS is a 42-item self-administered questionnaire designed to measure the severity of symptoms of depression, anxiety, and stress. Items are scored on a 4-point Likert scale. For each subscale (depression, anxiety and stress) a total score is computed by summing all individual items within that category. In addition, a DASS total score is calculated. Finally, a categorical score was calculated per subscale with five levels (normal, mild, moderate, severe, and extremely severe). The DASS had excellent psychometric properties, with high reliability and validity [64].

### 2.7. Pain Assessment

Self-reported pain was measured using a visual analog scale (VAS-pain). Similar to the VAS-craving, the VAS-pain was assessed on a horizontal line, of which the left end of the scale represented “no pain” and the right end “the most severe pain imaginable”. Participants quantified their current pain intensity by marking a point (from 0 to 100) on the line [65]. The VAS-pain was validated for CNCP patients, showing similar sensitivity compared with the often-used numeric rating scale (NRS) [66].

### 2.8. Procedure

After written informed consent, participants were planned for admission for opioid rotation to BuNa. Baseline measurements (T0) of COMM, VAS-craving, DASS, VAS-pain, OOS/SOS, and MINI-Plus were taken on the second day of admission, prior to any change in opioid use. During the rotation procedure, the measurements (OOS/SOS, VAS-craving and VAS-pain) were taken up to six times a day to facilitate dose titration of BuNa. Only the last measurement of the VAS-craving and VAS-pain before discharge (T1) was analyzed, in order to avoid state-dependent effects of the rotation on outcome measures. Two months post discharge follow-up measurements were performed (T2), including COMM, VAS-craving, DASS, and VAS-pain.

### 2.9. Statistical Analysis

In order to test our hypothesis, a per protocol analysis was used, given the small sample size and naturalistic explorative study design. Participants who still used BuNa at follow-up were considered completers. Descriptive data for continuous variables were presented as means and standard deviation (SD). Descriptive data for categorical variables were summarized by frequency and percentage. To answer our primary question, a univariate mixed model was performed with COMM scores as dependent variable, and time as a fixed factor (two levels). Subsequently, additional univariate mixed-model analyses were used to explore the effect of rotation to BuNa on craving (VAS-craving) and pain severity (VAS-pain), with time as a fixed factor with three levels. Finally, a multivariate mixed-model analysis was used to test the effects of BuNa rotation on the severity of psychological symptoms (DASS-depression, DASS-anxiety, and DASS-stress scores), with subscale (three levels) and time (two levels) as fixed factors. For all the mixed models, compound symmetry was used as covariance type. All statistical tests were carried out at the 0.05 level of significance using SPSS v.25 (IBM Corp, Armonk, NY, USA).

## 3. Results

**Figure 1:**
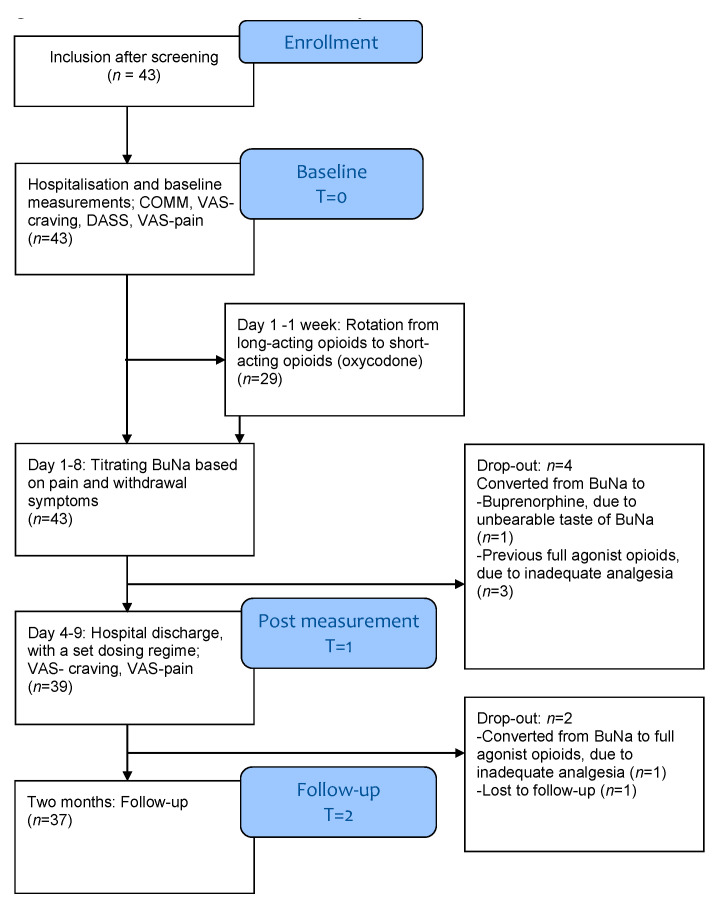
Consort flow chart of the study.

Thirty-seven of the 43 included participants finished the rotation and were included in the data analysis. Of the six participants who dropped out of the study, four were set back to their previous opioids due to inadequate analgesia, one was switched to buprenorphine instead of BuNa because of its bad taste, and one was lost to follow-up (Figure 1). As can be seen in Supplementary Table S1, the mean OME at baseline was higher in dropouts (593.3 ± 381.2) than in participants who completed the trial (328.3 ± 411.0; *p* = 0.015). No other significant differences were found between dropouts and completers (see Supplementary Table S1).

Table 1 shows demographical variables (gender, age, race, years of education, marital status and employment status). In addition, Supplementary Table S2 shows the psychiatric morbidity, as assessed with the MINI-plus, at baseline. The mean age of the participants was 47.5 years (±10.9) and 23 were male (62.2%). The mean OME at baseline was 328.3 mg (±411.0), the mean duration of the prescription opioids was 5.6 years (±3.8), and the mean daily dose of BuNa at discharge was 19.6 mg/4.9 mg (±8.2; ±2.1). The mean dose of BuNa at follow-up was 18.3 mg/4.6 mg (±9.9; ±2.5) per day. The main results of the descriptive statistics and the statistical analyses are listed in Table 2.

Primary outcomes:

After rotation from opioids to BuNa, opioid misuse, as indexed by the mean COMM score, decreased from 17.1 (SE = 1.40) at baseline, to 6.7 (SE = 1.45) at follow-up (F = 47.8; *p* < 0.000). Furthermore, the number of participants with current opioid misuse (COMM> 9) also decreased (pre: *n* = 29, 78.4%; post: *n* = 9, 24.3%; Chi-square = 19.2, *p* < 0.000). After rotation to BuNa, craving levels reduced over time from 39.3 (SE = 4.23) at baseline, to 21.6 (SE = 4.34) after rotation and 5.3 (SE = 4.34) at follow-up (F = 26.4, *p* < 0.000). Post hoc analyses showed a decrease in craving levels from baseline to the final day of admission (T0-T1: *p* < 0.000), and from baseline to follow-up (T0–T2: *p* < 0.000).

Secondary outcomes:

The severity of psychological symptoms on the DASS declined from baseline to follow-up (F = 56.3; *p* < 0.000). Post hoc analyses showed a decrease in all the subscales of the DASS (depression: F = 13.9; *p* = 0.001, anxiety: F = 23.6; *p* < 0.000, and stress: F = 14.1; *p* = 0.001). Lastly, self-reported pain on the VAS-pain reduced from 51.3 at baseline to 37.2 at follow-up (27.5% decrease; F = 3.28; *p* = 0.044). Post hoc analysis showed that this was mainly driven by an improvement in self-reported pain from baseline to follow-up (T0–T2: *p* = 0.013).

## 4. Discussion

The present study investigated the effects of rotation from full mu-opioid receptor agonists to BuNa, in patients with CNCP and OUD, on (i) current opioid misuse, (ii) opioid craving, (iii) psychiatric symptoms, and (iv) self-reported pain. Thirty-seven of the 43 participants finished the trial. Patients dropping out was mainly due to the inadequate pain control of BuNa (four out of six participants). As hypothesized, the opioid rotation to BuNa reduced current opioid misuse and opioid craving, and improved psychiatric symptoms and self-reported pain. This suggests that opioid rotation to BuNa could have beneficial effects in patients with CNCP and OUD.

Our findings are in compliance with previous research, suggesting that buprenorphine (with or without naloxone) reduces opioid misuse and craving in people with OUD [23,34,35,36,37,38,39,67,68]. To our knowledge, only one study explored the effects of BuNa on craving in patients with CNCP and OUD, showing a significant reduction of approximately 45 points on the VAS-craving scale [23], in line with the observed reduction in craving observed in the current study. However, it should be noted that this previous study (i) had a smaller study population (*n* = 19), (ii) with participants showing higher baseline craving and (iii) could not distinguish between methadone and BuNa rotation. Furthermore, it should be noted that about one fourth of participants still exceeded the cut-off value for opioid misuse on the COMM at follow-up. This may indicate that after BuNa OAT some patients may still misuse opioids. Future studies should address how to support these patients in gaining control over their opioid use. In addition, future research might consider a broader assessment of opioid misuse. For instance, using biomarkers for the use of opioids (urine testing), or DSM-5 criteria for OUD.

A possible explanation for the observed reduced misuse and craving of opioids after rotation to BuNa might be due to the slower dissociation rate of buprenorphine, compared to most full mu-opioid receptor agonists [32]. Furthermore, it has been suggested that the partial agonism of buprenorphine in the mu-opioid receptor might cause less severe withdrawal symptoms [69]. Since the naloxone component in BuNa has limited availability in the central nervous system due to its first-pass effect, it is unlikely that the current sublingual administration of naloxone contributed to the observed effects on craving [70]. However, any contributing central effects of naloxone cannot be fully ruled out.

The beneficial effects of rotation to BuNa on depression, anxiety and stress, as observed in the current study, are also in line with previous research, showing antidepressant properties of buprenorphine in patients with depression [45,51], in patients with OUD [45,47,48,49,50], and in patients with OUD and CNCP [23]. It has been hypothesized that the kappa-antagonism of buprenorphine might contribute to its antidepressant and stress-reducing effects [71,72]. Indeed, the brain kappa-opioid receptor system has been linked with several psychological symptoms, including depression and anxiety, and stress-related symptoms in addictive disorders [73]. Furthermore, the anti-depressant and stress-reducing effects of kappa-antagonists have also been observed in several animal studies [74,75]. In addition, the discontinuation of full mu-opioid receptor agonists might have contributed to the observed beneficial effects of BuNa rotation on mood symptoms. Indeed, full-mu receptor agonists have been shown to have depressogenic properties [76,77,78]. The present study also supports previous observations of BuNa as potentially beneficial for pain management in CNCP patients with OUD. The reduction in pain intensity observed here (VAS -13 points on average), is comparable with previous studies on the analgesic properties of buprenorphine in CNCP patients with OUD (change in VAS ranging between −8 and −45) [23,30,31] and in CNCP patients without OUD (change in VAS ranging between −23 and −37) [27]. It has been hypothesized that the partial mu-opioid agonism and/or kappa-opioid antagonism might reduce pain perception, due to a reduction of opioid-induced hyperalgesia [79,80]. The chronic use of full mu-opioid receptor agonists has been shown to cause hyperalgesia, associated with increased sensitization of the central nervous system for pain stimuli, resulting in lower pain thresholds and higher pain perception [13,14]. Reductions of opioid-induced hyperalgesia after BuNa rotation may thus improve analgesia in CNCP patients previously treated with full mu-opioid receptor agonists. However, due to the unreliable OME conversion rates of BuNa, it cannot be ruled out that participants used a higher OME after switching to BuNa. This may have contributed to the observed overall reduction in pain severity.

It should be mentioned that, since four patients dropped out due to inadequate analgesia, the analgesic effects of BuNa rotation may be overestimated in the current study. In addition, participants who dropped out of the study showed higher baseline OME levels in comparison with the analyzed participants. This may indicate that participants with a higher OME at baseline are more likely to fail the conversion to BuNa. As can be seen in Supplementary Table S1, those who dropped out did also have non-significant (i) higher baseline craving levels, (ii) a younger age, and (iii) a higher baseline VAS-pain. Future studies should further explore which patients are most likely to benefit from rotation to BuNa, to facilitate patient–treatment matching.

The current findings should be interpreted in the light of several study limitations. Due to the relatively small study population (*n* = 37) and observational design, generalization of the study results is limited and prevents drawing firm conclusions regarding the effectivity of BuNa as OAT for patients with CNCP and OUD. Furthermore, with a follow-up period of two months, our study did not investigate the long-term potential of BuNa. Future randomized studies, with a larger study population, longer follow-up period and a control group, are necessary to confirm our findings. In addition, our study population consisted of mainly males (62.2%) and patients with nociceptive pain (54.1%). Future studies should evaluate whether our findings are also apply to females and patients with non-nociceptive pain.

It should be noted that opioids should preferably be discontinued in patients with CNCP. Some studies suggest that opioid tapering might be beneficial in CNCP patients [81]. In methadone-rotated participants, success rates were about 28% [82]. Though this was not the aim of the current study, it was highly relevant to investigate the tapering of BuNa in patients with CNCP and OUD.

Finally, future research should also explore the beneficial effects of non-pharmacological interventions in patients with CNCP and OUD, for instance in addition to BuNa rotation. Cognitive Behavioral Therapy and Mindfulness are effective in reducing pain [83,84,85] and addictive behaviors, including OUD [86]. Therefore, additional psychotherapy may increase the effectiveness of rotation from full agonist opioids to BuNa in patients with CNCP and OUD and facilitate tapering BuNa after the rotation process.

In conclusion, this prospective observational study showed that the rotation from full mu-opioid receptor agonists to BuNa had beneficial effects on opioid misuse, opioid craving, the severity of co-occurring psychiatric symptoms, and the pain perception of patients with CNCP and OUD. These findings suggested that BuNa rotation might be a valuable strategy in CNCP patients with OUD. Future studies should replicate these findings, and explore which patients benefit the most from rotation to BuNa, as 14% of our participants dropped out of the trial, mainly due to inadequate analgesia.

## Figures and Tables

**Table 1:** Participants characteristics.

	Total (*n* = 37)
Marital status	
Single	8 (21.6%)
Cohabitating	29 (78.4%)
Education ^‡^	
Low	3 (8.1%)
Middle	25 (66.7%)
High	9 (24.3%)
Employment ^†^	10 (27.0%)
Substance use ^§^	
Nicotine	14 (37.8%)
Alcohol	15 (40.5%)
Drugs	10 (27.0%)
Type of pain ^‡‡^	
Nociceptive	20 (54.1%)
Neuropathic	13 (35.1%)
Idiopathic	4 (10.8%)
Type of opioid ^††^	
Oxycodone	28 (75.7%)
Methadone	6 (15.8%)
Fentanyl	10 (27.0%)
Other	9 (24.3%)
Multiple opioids	14 (37.8%)
Other analgesics ^††^	
Paracetamol	21 (56.8%)
NSAID	4 (10.8%)
Antidepressant	10 (27.0%)
GABA/glutamatergic (e.g., pregabalin)	15 (40.5%)
Other medication ^††^	
Sedatives	10 (27.0%)
Psychotropics	12 (32.4%)
Gastrointestinal	21 (56.8%)
Laxatives	14 (37.8%)
Cardiac	9 (24.3%)
Pulmonary	6 (16.2%)

Living with a partner; ^‡^ highest achieved degree of education (low: no education, pre-primary, primary, lower secondary education, compulsory education, and initial vocational education; middle: upper secondary general education, basic vocational education, secondary vocational education, and post-secondary education; high: specialized vocational education, university/college education, and doctorate and equivalent degrees); ^†^ Percentage employed; ^§^ Amount of participants answering “yes’’ on substance use over the past 30 days; ^‡‡^ Main cause of the chronic pain; ^††^ Amount of participants taking these types of medication.

**Table 2:** Mean values of the dependent variables, combined with the corresponding *p*- and F-values.

	Baseline; Mean (±SE; 95% CI; Z-Score)	Discharge; Mean (±SE; 95% CI; Z-Score)	Follow-up; Mean (±SE; 95% CI; Z-Score)	F-Value (df)	*p*-Value
COMM	17.1 ± 1.40 (14.3–19.9; 0.504)	-	6.7 ± 1.45 (3.8–9.6; −0.539)	36.50 (35.61)	0.000 *
VAS-craving	39.3 ± 4.23 (30.5–48.2; 0.590)	21.4 ± 4.34 (12.7–30.0; −0.016)	5.3 ± 4.34 (−3.4–13.9; −0.557)	26.42 (70.75)	0.000 *
DASS	12.1 ± 1.17 (9.8–14.5; 0.296)	-	6.6 ± 1.19 (4.2–9.0; −0.341)	56.32 (173.49)	0.000 *
VAS-pain	51.3 ± 4.53 (41.6–59.6; 0.267)	41.7 ± 4.42 (33.0–50.5; −0.057)	37.2 ± 4.42 (28.5–46.0; −0.222)	3.28 (70.11)	0.044 *

SE, standard error; CI, confidence interval; VAS, visual analog scale; COMM, current opioid misuse measurement; DASS, depression anxiety stress scale; * *p*-value considered statistically significant (*p* < 0.05).

## Data Availability

The data of the current study will not be made publicly available, but can be made available upon request to the authors.

## Supplementary Materials

The following are available online at https://www.mdpi.com/article/10.3390/jcm10163727/s1, Table S1: Differences in mean between analyzed participants (*n* = 37) and drop-outs (*n* = 6); Table S2: Baseline psychiatric comorbidity as defined with the Mini International Neuropsychiatric Interview (MINI-plus).
