# Supplementary material for: Beneficial Effects of Opioid Rotation to Buprenorphine/Naloxone on Opioid Misuse, Craving, Mental Health, and Pain Control in Chronic Non-Cancer Pain Patients with Opioid Use Disorder
Source: J Clin Med. 2021 Aug 21;10(16):3727. doi: 10.3390/jcm10163727 (PMC8396821; doi:10.3390/jcm10163727)
Supplement: Supplementary file 1 [file jcm-10-03727-s001.zip › jcm-1317416-supplementary.pdf]

Table S1. Differences in mean between analyzed participants (n = 37) and drop-outs (n = 6).

	Analyzed † - mean ±SD	***Drop outs - mean ±SD***	***p-value¶***
Age	47.5 ± 10.9	39.3 ± 12.8	0.150
OME ‡	328.3 ± 411.0	593.3 ± 381.2	0.015 *
VAS-pain §	52.1 ±25.3	61.3 ± 7.4	0.667
VAS-craving §	40.8 ±31.5	48.7 ± 44.6	0.591

OME, oral morphine equivalent; VAS, visual analogue scale; † participants who finished the trial; ‡ OME at baseline; § VAS baseline; ¶ analyzed using Mann-Whitney U test; * p-value considered statistically significant (p<0.05).

Table S2. Baseline psychiatric comorbidity as defined with the Mini International Neuropsychiatric Interview (MINI-plus).

	**Total¶ (N = 37)**
Total prevalence ‡	23 (62.2%)
Depression	13 (35.1%)
Dysthymia	1 (2.7%)
(hypo)mania	0
Anxiety disorder	4 (10.8%)
Agoraphobia	5 (13.5%)
Social phobia †	3 (8.1%)
Obsessive disorder	1 (2.7%)
Compulsive disorder	1 (2.7%)
OCD	1 (2.7%)
PTSD†	2 (5.4%)
Alcohol use disorder	2 (5.4%)
Substance use disorder	3 (8.1%)
Generalized anxiety disorder †	1 (2.7%)
ADHD	4 (10.8%)

OCD, obsessive–compulsive disorder; PTSD, post traumatic stress disorder; ADHD, attention deficit hyperactivity disorder; ¶ The life time prevalence of psychiatric comorbidities according to the MINI-plus; ‡ The amount of participants that met the criteria of at least one comorbid mental health disorder; † The current prevalence of psychiatric comorbidities according to the MINI-plus.
